# New evidence on the tool-assisted hunting exhibited by chimpanzees (*Pan troglodytes verus*) in a savannah habitat at Fongoli, Sénégal

**DOI:** 10.1098/rsos.140507

**Published:** 2015-04-15

**Authors:** J. D. Pruetz, P. Bertolani, K. Boyer Ontl, S. Lindshield, M. Shelley, E. G. Wessling

**Affiliations:** 1 Department of Anthropology, Iowa State University, Ames, IA 50011, USA; 2 Ecology and Evolutionary Biology program, Iowa State University, Ames, IA 50011, USA; 3 Department of Statistics, Iowa State University, Ames, IA 50011, USA; 4 Department of Political Science, Iowa State University, Ames, IA 50011, USA; 5 Leverhulme Centre for Human Evolutionary Studies, University of Cambridge, Cambridge, UK; 6 Department of Social Sciences, Michigan Technological University, Houghton, MI 49931, USA; 7 Department of Primatology, Max Planck Institute for Evolutionary Anthropology, Deutscher Platz 6, 04103 Leipzig, Germany

**Keywords:** chimpanzee, hunting, tool use, Sénégal, savannah

## Abstract

For anthropologists, meat eating by primates like chimpanzees (*Pan troglodytes*) warrants examination given the emphasis on hunting in human evolutionary history. As referential models, apes provide insight into the evolution of hominin hunting, given their phylogenetic relatedness and challenges reconstructing extinct hominin behaviour from palaeoanthropological evidence. Among chimpanzees, adult males are usually the main hunters, capturing vertebrate prey by hand. Savannah chimpanzees (*P. t. verus*) at Fongoli, Sénégal are the only known non-human population that systematically hunts vertebrate prey with tools, making them an important source for hypotheses of early hominin behaviour based on analogy. Here, we test the hypothesis that sex and age patterns in tool-assisted hunting (*n*=308 cases) at Fongoli occur and differ from chimpanzees elsewhere, and we compare tool-assisted hunting to the overall hunting pattern. Males accounted for 70% of all captures but hunted with tools less than expected based on their representation on hunting days. Females accounted for most tool-assisted hunting. We propose that social tolerance at Fongoli, along with the tool-assisted hunting method, permits individuals other than adult males to capture and retain control of prey, which is uncommon for chimpanzees. We assert that tool-assisted hunting could have similarly been important for early hominins.

## 2. Introduction

Palaeoanthropologists use nonhuman primate models as well as living humans in addition to the fossil record to illuminate aspects of extinct hominin behaviour [1–4]. For example, tool-using primates have been used to provide insight into the potential tool-using capabilities of early hominins that predate lithic technology [1,2]. Similarly, anthropologists have traditionally used apes as analogous models to better understand the role of hunting in human evolution, using the argument from homology that closely related species are likely to show similar adaptive ‘solutions’ to evolutionary ‘problems’ [1,2,5–7]. Additionally, in an evolutionary context, derived traits relative to the ancestral condition are considered the most informative for posing hypotheses [8]. However, our understanding of what is unique to the human lineage continues to be redefined as we learn more about other species, and similarities with other animals must be closely examined to understand wherein these differences lie [9].

Chimpanzees hunt and share meat at every site where they have been studied over extended periods of time [5–7,10,11], and although meat does not make up a large proportion of their diet [7] it is considered a valued resource [5,11,12]. Like humans, chimpanzees exhibit sex differences in hunting behaviour, with males hunting more than females [5,6,13–15]. Although in some human societies women's hunting contributes significantly to calories brought in via hunting [15], and their hunting styles are considered more efficient than men's in some cases [16], human males are also typically regarded as the most important hunters in a group or population [5,6,13–15]. Most theories suggest that hunting by early hominins was also primarily an adult male activity [5,7]. Understanding these apparent similarities in male Hominoid hunting strategies can assist anthropologists in discerning whether these similarities are examples of homoplasy or have evolutionary significance. Moreover, elucidating variation in what is presented as a general pattern of male-biased hunting [5] provides a better picture of what some anthropologists consider to be the basal hominin condition [17,18].

Here, we present data on the tool-assisted hunting behaviour of chimpanzees in a woodland-savannah environment and consider the relevance of their behaviour to hominin evolution by using a relational type of analogous model (*sensu* [3]) by comparing them with forest-dwelling chimpanzees. This may elucidate the influence of the savannah environment on the behaviour of apes (e.g. a non-trivial analogy *viz*. [2]), as the environment at Fongoli is ecologically similar to that of the earliest known hominins [19].

**Figure 1.**
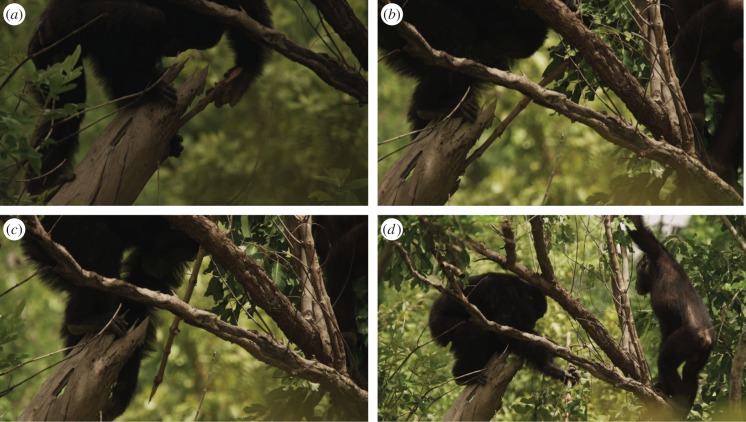
Tool-assisted hunting by chimpanzee at Fongoli, Sénégal. Adult male chimpanzee uses tree branch with modified end to (*a*–*c*) stab into a cavity within a hollow tree branch that houses a *Galago* he ultimately captures as (*d*) his adolescent brother looks on. Images are courtesy of BBC.

Preliminary data on tool-assisted hunting indicated that sex differences characterize this behaviour, with female chimpanzees more often hunting *Galago senegalensis* prey within tree cavities using a jabbing tool [20] (figure 1*a*–*d*). In addition to examining the age–sex class differences in *Galago* hunting behaviour, we compare these differences to other hunting behaviours at Fongoli where tools were not used. We use our results in a referential model to inform hypotheses regarding the evolution of hunting behaviour in hominins.

## 3. Material and methods

The Fongoli chimpanzee community ranges within the Kedougou Region in southeastern Sénégal (12^°^40′ N, 12^°^13′ W). The savannah environment here is a mosaic of woodland, grassland, bamboo and gallery forest habitats [3,18,20]. The extensive dry season lasts over seven months, and rainfall usually averages less than 1000 mm annually [21]. Maximum temperature in the dry season exceeds 40^°^C.

The Fongoli community averaged 31.7 individuals annually (range 29–36) from 2005 to 2014. Most apes were habituated by 2005, when systematic behavioural observation began, but some adult females remained only semi-habituated for several years, in that they exhibited signs of nervousness around observers when adult males were absent. The first tool-assisted hunts were recorded in 2005. Not all adult females were identified until early 2006. Owing to the rare but real risk of poaching females for infants for the pet trade, only adult males are focal subjects [22]. When females were in mixed-sex groups, data on their hunting behaviour were collected. We expect that the focus on adult males as focal subjects underestimates adult female hunting behaviour. Age–sex classes were defined as [23]: infants age 4 years or less, juveniles 4–7 years, adolescents more than 7 and less than 15 years (males) or until a female gave birth, and adults as males more than 15 years.

### 3.1 General vertebrate hunting behaviour by Fongoli chimpanzees

A hunting bout was defined as an observed *capture* or *possession* of a mostly intact prey item by a chimpanzee. Specifically, categories were defined as: (i) capture—where the hunt was visually observed; (ii) capture out of sight—where the capture was not observed, but the prey was obtained immediately after a chase was observed or, in the case of tool-assisted hunting, when the hunting behaviour was heard (i.e. tools being jabbed into hollow cavities), but the actual capture was not seen; or (iii) possession—when a chimpanzee had control of an entire or mostly entire carcass but the capture was not observed. Of the 47 cases classified as possession, several involved only a missing head (*n*=3), but we also recorded some cases of prey missing the head and one or more limbs (*n*=5). In almost all cases, prey items except bushbuck fawns (*Tragelaphus* *scriptus*) were consumed headfirst.

One assumption that we made regarding possession as indicative of hunting is that observers would find hunters and prey before much of the carcass was eaten. Monkey and bushbuck hunts are often accompanied by ‘meat barks’ [24] that alert observers to the behaviour and, in some cases, prey species give alarm calls (e.g. vervets (*Chlorocebus aethiops*) and baboons (*Papio hamadryas papio*)). In the case of tool-assisted hunting, the bout is often detected via the audible sound of a tool jabbed into a hollow trunk or limb. During monkey hunts, observers often lagged behind chimpanzees either purposefully (during stealth hunts, so as not to alert the prey species), or because it was difficult to keep up with rapidly moving chimpanzee hunters. Therefore, hunting effort data (e.g. failed hunts) for non-tool-assisted hunting are sparse in terms of individuals involved and are not analysed here, as they are not considered comparable to the data available on effort (i.e. failed hunts) recorded for tool-assisted hunting (see below).

It is possible that, in rare cases, a dominant individual immediately took prey from a subordinate, and we would thus incorrectly identify the captor, but such theft appears rare at Fongoli. Thus far, we have observed theft five times only, with two more possible cases of theft, out of 99 total captures of individual prey animals. Four of these cases involved alpha male theft of the prey item. Another case involved a mid-ranking adult male taking a baboon carcass from an adult female. Additionally, the current alpha male at Fongoli may have also taken a bushbuck carcass from an adult female, and a juvenile vervet monkey from an adolescent female. However, in the latter two cases, this interpretation was based on the observer's impression of individual chimpanzees' behaviour, such as tantrum screaming and intense following by the females, but not visual confirmation. We scored these five cases of theft according to the initial captor except in the latter two cases of possible theft when the alpha male was scored as having primary possession of the prey. In one additional case, an older juvenile female caught a *Galago* by hand but did not kill it. Her mother approached and took the prey, without any protest from her daughter, killed it and shared the meat with her daughter. We scored the daughter as the captor in this case.

### 3.2 Tool-assisted hunting by Fongoli chimpanzees

Whenever a tool-assisted hunting bout (as described in [20]) was detected the observer moved to within 20 m of the hunter. We categorize the method that Fongoli chimpanzees use to capture *Galago* prey as hunting following the terminology used in the first incident of similar behaviour reported in Tanzania, where a subadult female used a stick tool to rouse a squirrel from a hollow [25]. Additionally, we consider this hunting because the prey is mobile rather than sedentary, the prey can be aggressive, and regardless of size, Fongoli chimpanzees show aversion to being bitten by a *Galago*.

Variables collected during hunting bouts included individual identity, age and sex of the hunter. Cases where individuals possessed a *Galago* but were not observed to capture it (*n*=24), or when they captured it without the aid of a tool (*n*=12), were not included in analyses of tool-assisted hunting but were included in the larger database of overall hunting at Fongoli. The 22 original bouts of tool-assisted hunting reported [20] are included in this study. Only bouts where subjects were positively identified (*n*=294) were used in subsequent generalized linear mixed model (GLMM) analyses.

The number of individuals in each age–sex class on days when any chimpanzee was observed to hunt with tools was averaged over the study to calculate expected tool-assisted hunting values for each age–sex class based on their abundance within the group on these days. These hunting days were taken as representative of age–sex class availability for this analysis given that the Fongoli chimpanzee community is more cohesive than those at other sites [26], with all individuals usually ranging together during the transitional (dry to wet) and wet seasons [27] when 95% of tool-assisted hunts were recorded (see below). In most analyses, infant and juvenile data were combined since their skill was judged as ineffective for prey capturing, and no successful hunt was ever recorded for them. A successful hunt was defined as when an individual captured a *Galago* with the use of a tool. Of the 35 individuals seen to hunt with tools, most had been in the study group and at least 2 years of age (the earliest age when individuals were recorded to hunt with tools) for the majority of the study. On average, individual male chimpanzees that were recorded to hunt with tools (*n*=19) were present for 8 years (range 3–10 years) of the 10-year study, while females (*n*=16) were present for 7 years on average (range 1–10 years).

### 3.3 Statistical analyses

We used the mean number of individuals present in the eight different age–sex classes on days on which chimpanzees were observed to hunt with tools (i.e. hunting days) to calculate the expected values for tool-assisted hunting for each age–sex class. We used annual values averaged over the course of the study to calculate expected values for male versus female hunting behaviour in general. The type I (alpha) error level for declaring a result to be statistically significant was set at less than or equal to 0.001 following Colquhoun [28], to guard against false positive findings.

To test whether tool-assisted hunting probability differed between females and males, a GLMM model [29] was estimated with binomial error structure and logit link function whereby the response was the sex of the hunting individual (female=0; male=1). The model included no fixed effects and only the random intercept of subject. To account for the varying proportion of males in the group, we included this proportion (log-transformed after dividing by the mean proportion of males) as an offset term [30] into the model. The only term of interest on this model is the intercept. If this term is statistically significant then the probability for males (and females) to hunt differs from chance probability (given the respective proportion of male and female group members). This model was fitted using the function glmer of the R package lme4 [31], and the sample size was 238 tool-assisted hunting bouts by 32 individuals. A smaller sample size was analysed due to the lack of complete demographic data for all days on which hunts were recorded. An additional model was estimated in which age class (coded as described above) was included as a fixed effect to evaluate whether hunting probability of males and females differed among age classes.

To test whether age class or sex had an influence on hunting success, a GLMM model was estimated with binomial error structure and logit link function. In this model, we included age, sex and their interaction as fixed effects and subject as a random effect. Prior to estimating the model we coded age class as 1 (infant), 2 (juvenile), 3 (adolescent) and 4 (adult) and then *z*-transformed the variable to a mean of zero and a standard deviation of one. We fitted the model in R using the function glmmadmb of the package glmmADMB [32,33]. It was not possible to include a random slope [34,35] for age because about half of the individuals appeared in only one age class over the 10-year period. As an overall test of the significance of the fixed effects as a whole [36] the full model was compared with a null model that comprised only the random effect of individual using a likelihood ratio test [37]. Individual effects were assessed using likelihood ratio tests comparing the full model against a reduced model lacking the term to be tested. The sample size for this model was 294 hunting bouts of 35 individuals. This model revealed some moderate complete separation issues [38], because of which we do not show estimates and their standard errors.

## 4. Results

### 4.1 General vertebrate hunting behaviour by Fongoli chimpanzees

**Figure 2.**
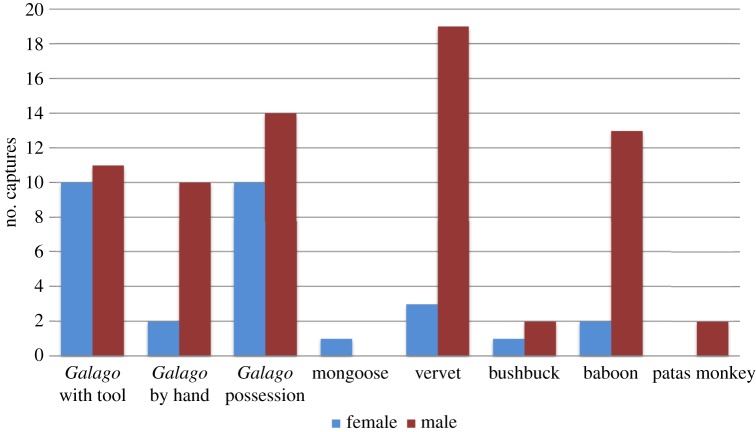
Sex differences in prey items obtained during tool and non-tool-assisted hunting at Fongoli.

A total of 99 hunting cases (including tool-assisted and non-tool-assisted hunting) were recorded during the study. Of these, 41 were visually confirmed captures, 50 were scored as possession and eight were scored as captured immediately out of sight. Females accounted for almost one-third (30%; *n*=28 cases), while males accounted for 70% (*n*=71) of all captures. When these values were analysed according to males' and females' representation in the chimpanzee community over the study period (57% male, 43% female), the difference in prey capture was significant (*χ*^2^=18.6766, d.f.=2, *p*<0.001). Tool-assisted *Galago* captures accounted for 21% of all hunting cases, and both males and females preyed on *Galago* more than any other vertebrate species. Over half of all vertebrates recorded to be captured by Fongoli chimpanzees were *Galago* (figure 2).

Chimpanzees were observed to run down or grab *Galago* prey without the use of a tool in 12 cases (nine adult male, one adult female, one adolescent male, one adolescent/late juvenile female). In another 24 cases, the capture was not observed, so it is unknown as to whether these were tool-assisted hunts or were captures by hand. Thirteen of these *Galago* capture cases were by adult males, 10 by adult females, and one by an adolescent male. In all, adult males accounted for just over one-half of all *Galago* prey obtained (35 of 67 cases), regardless of the capture method used.

Males and females varied in the distribution of their vertebrate prey species (figure 2). While the prey profile for females was dominated by *Galago* (75%), this same species accounted for 47% of the male chimpanzee profile. Males' profile included more vervets (27%) and baboons (18%) than did the profile for females (figure 2). Only males included patas monkeys (*Erythrocebus patas*) in their captures, while only females captured the banded mongoose (*Mungos mungo*). Both males (*n*=2) and females (*n*=1) were seen with bushbuck fawns, which were also the largest vertebrate prey item recorded for Fongoli chimpanzees. Most monkeys captured were immature individuals, while most *Galago* prey appeared to have been adults.

Individual chimpanzees varied in their prey capture behaviour (table 1). About 40% of all successful hunters were female. The most successful hunter was the oldest and lowest-ranking adult male. Two high-ranking adult females were among the top 10 hunters at Fongoli, and many hunters were recorded with only one to two captures over the course of the entire study period. Two males who had been alpha were also among the top 10 hunters at Fongoli, although another alpha was recorded as having captured prey only once.

**Table 1.** Individual Fongoli chimpanzee hunting success according to vertebrate prey species. In some cases, an individual was in more than one age class when they captured prey. Cases in which these individuals captured prey as an adolescent are indicated in parentheses.

rank	subject	age class at time of prey capture	sex	*Galago*	vervet	baboon	bushbuck	patas monkey	mongoose	total captures
1	Siberut	adult	male	5	4	3	1	1		14
2	Lupin	adult	male	11	1					12
3	Bilbo	adult	male	2	5	2				9
4	Tumbo	adult, (adolescent)	female	4 (2)	1	1				8
5	Bo	adult, (adolescent)	male	3	2 (1)			1		7
5	David	adult	male	1	3	2	1			7
7	Farafa	adult	female	5			1			6
8	Bandit	adult	male	2	3					5
8	K.L.	adult	male	2		3				5
10	Luthor	adult	male	4						4
11	Lily	adult, (adolescent)	female	1 (1)						2
11	Tia	adult	female	1					1	2
11	Nene	adult	female	2						2
11	Lucille	adult	female	2						2
11	Diouf	adult	male	1		1				2
16	Karamoko	adult	male			1				1
16	Yopogon	adult	male			1				1
16	Fanta	adolescent	female	1						1
16	Foudouko	adult	male	1						1
16	Frito	adolescent	male	1						1
16	Jumkin	adult	male	1						1
16	Mike	adult	male	1						1
16	Natasha	adult	female	1						1
16	Sounkaro	juvenile	female	1						1
16	Sonja	adolescent	female		1					1

### 4.2 Tool-assisted hunting by Fongoli chimpanzees

A total of 308 tool-assisted hunts were recorded over the course of the study, with individual chimpanzees identified in 294 cases. Almost all hunts occurred during the wet season (June–September) or the transitional months (May, October), while only 15 (4.9%) hunts were recorded during the dry season (November through April). During the 10-year study, the annual average was 30.8 hunts per year (range 9–61).

**Figure 3.**
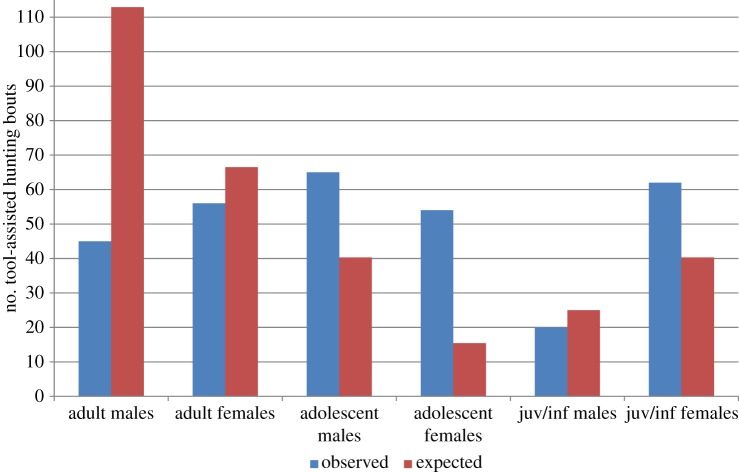
Observed versus expected tool-assisted hunting frequency in chimpanzee age–sex classes at Fongoli, Sénégal. Expected frequencies are calculated by multiplying the % total hunting frequency by the % of that age–sex class comprising the average chimpanzee party recorded on days on which tool-assisted hunting was recorded. Based on 302 tool-assisted hunts where age–sex class was known. Adol, adolescent; juv/inf, juvenile and infants.

Both males and females at Fongoli engaged in tool-assisted hunting, with females recorded to hunt more with tools (*n*=175 times) than males (*n*=130 times; figure 3). Males were much less likely to hunt than females, accounting for the proportion of males (61%) and females (39%) comprising parties on days that chimpanzees were recorded to hunt with tools (estimate±s.e.=12.57±2.13, *z*=5.908, *p*<0.001). In fact, while group composition on hunting days included an average of 61% males, only 39% of the hunts were by males.

Thirty-five different individuals were observed to hunt with tools, out of a potential 44 chimpanzees known during the course of the study period. Almost all Fongoli chimpanzees over the age of two have been recorded to hunt with tools over the years, and the median number of hunts per individual is approximately 11 (range 1–27 [27]). Overall, female chimpanzees averaged 10.6 hunts each (range 2–27), while males hunted on average 6.8 times each (range 1–22). Five of the individuals who were never observed to hunt disappeared early in the study, and two are currently young infants (less than 2 years of age). Therefore, only two individuals that we have had reasonable expectations of observing to hunt with tools have not yet been recorded to engage in this behaviour.

When individuals were assigned to one of six age–sex classes, adolescent males hunted most frequently (*n*=65), followed by juvenile/infant females (*n*=62), adult females (*n*=56), adolescent females (*n*=54), adult males (*n*=45) and juvenile/infant males (*n*=20). However, when including age class in the model, we did not find that male hunting probability significantly differed among age classes (estimate±s.e.=0.44±1.81, *χ*^2^=0.06, d.f.=1, *p*=0.809). Variance was high in terms of overall tool-assisted hunting frequency and when hunting frequency was analysed according to age–sex class, thereby demonstrating the high level of variability among individual hunters (table 2).

**Table 2.** Age class summary statistics: tool-assisted hunting.

	overall hunting	adults	adolescents	juveniles	infants
number of hunts	295	96	111	56	32
number of individual hunters	35	21	14	10	10
mean±s.d. hunts per individual	8.429±6.559	4.571±4.214	6.529±5.064	3.733±4.728	2.909±2.212
median hunts per individual	7	3	6	3	3
range of hunts per individual	1–27	1–15	1–20	1–18	1–7

**Figure 4.**
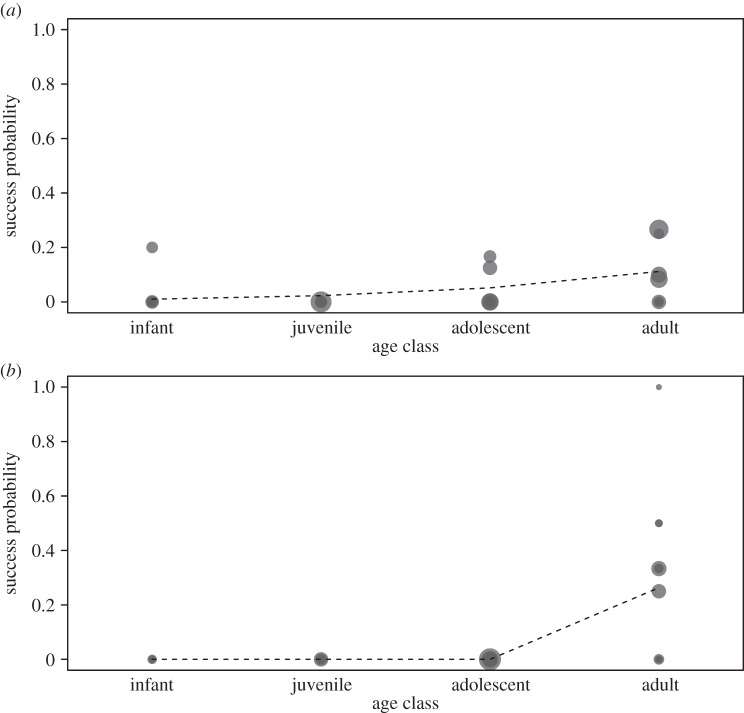
Probability of hunting success as a function of age, separately for (*a*) females and (*b*) males. Each individual per age class is depicted by one point, whereby the area of the points corresponds to the sample size (range 1–20). The dashed lines depict the fitted probability (derived from models estimated separately for females and males).

The overall success rate of tool-assisted hunting was low when individuals were pooled (7.5%, *n*=308 hunts), but certain age–sex classes were more successful than others. Juveniles and infants were never observed to capture *Galago* prey, and when they are excluded from analyses the success rate rose to 10.3% (*n*=213 hunts) for adolescent and adult hunters. When only adults are considered, the success rate increased to 20.6%, with males (22%, *n*=46 hunts) having slightly greater success than females (20%, *n*=56 hunts). Adolescent females exhibited a success rate of 4% (*n*=54 hunts), while adolescent males had a 1.5% success rate when they hunted with tools (*n*=64). Overall, there was an impact of sex, age and their interaction on hunting success (full null model comparison: *χ*^2^=27.36, d.f.=3, *p*<0.001). The probability of successful hunting increased with age and more so in males, although not significantly so (*χ*^2^=9.74, d.f.=1, *p*=0.002; figure 4).

## 5. Discussion

Savannah-dwelling chimpanzees at Fongoli, Sénégal, are the only non-human population known to systematically hunt vertebrate prey with tools [20]. We analysed 308 such hunts to test the hypothesis that significant age–sex class differences exist regarding this behaviour, based on our preliminary report [20], and we compared these results to the pattern of overall hunting at the site. Adult males were less likely to engage in tool-assisted hunting behaviour although they were the more successful hunters at Fongoli overall. Given the common view that adult males are the primary hunters at sites where chimpanzees have been studied elsewhere [1,2,4,5], the pattern at Fongoli is intriguing. Additionally, even though adult males were relatively successful at tool-assisted *Galago* hunting, females were equally successful, and other age–sex classes accounted for more than one-third of all *Galago* prey captures, which is unexpected [1]. Why is such hunting prevalent at Fongoli and not elsewhere? With regard to tool-assisted hunting, a number of explanations might be posed to explain why adult males do not hunt with tools as often as females.

The savannah environment might be viewed as the catalyst for tool-assisted hunting by savannah chimpanzees in Sénégal in that apes here exploit prey that is largely ignored by forest-dwelling chimpanzees [1,3,6,39]. Chimpanzees' preferred prey at other sites, the red colobus monkey (*Piliocolobus* *badius*) [1,6], is absent in the dry, open savannah-woodland at Fongoli. *Galago* prey is the most common vertebrate captured by Fongoli chimpanzees and accounts for most prey captured by females, almost always via tool use, but less than half of prey captured by males at Fongoli, who use tools but also chase down *Galago* prey flushed by other individuals using tools. One explanation for the underrepresentation of males in tool-assisted hunting might involve the relatively small return on their effort given the size of *Galago* prey compared to other prey species they capture at Fongoli (i.e. *E. patas*, *C. aethiops*, *P. hamadryas papio*) but, in terms of number of prey items, *Galago* prey also accounts for the top vertebrate prey item in Fongoli male chimpanzees' diet. Since their capture of *Galago* prey often occurs after other individuals flush them from their sleeping cavities with tools, much of males' hunting of this particular prey species could be described as opportunistic rather than targeted. The energetic expense as well as other potential costs associated with hunting monkey prey compared to *Galago* (but see [40]) would appear to offset a relatively poor return.

Perhaps our results should not be surprising, however, given that tool use proficiency in chimpanzees and bonobos (*Pan* *paniscus*) has been interpreted as being more heavily female-oriented [5,16,25,41]. While such stereotypical behaviour as female chimpanzee termite fishing might appear analogous to tool-assisted hunting, the similarities are superficial in that the prey is hidden from view, and a tool is used to acquire it. The behaviour of the prey, size/return per unit time of prey and activity required of the hunter or fisher are distinctly different. Additionally, vertebrate meat is considered a preferred food for chimpanzees [6]. Perhaps a better question is why individuals other than males at other chimpanzee study sites do not hunt more. Although meat sharing occurs at virtually every site where chimpanzees hunt vertebrates, the tendency for dominant males to monopolize carcasses seems to be the norm (or at least emphasized in the literature [1]), with an average of 25% of captured prey being taken by more dominant individuals [4,42]. Theft at Fongoli, by contrast, was less than 5% of all captures. Thus, it may not pay for low-ranking individuals, such as females and immatures, to hunt as frequently at other sites where chimpanzees have been studied. However, West African chimpanzees in general and those at Fongoli specifically are more cohesive in their ranging and grouping behaviour than elsewhere [24], so that data on female hunting are lacking for another reason (e.g. females are not studied as extensively as males).

Ultimately, the explanation for the pattern of tool-assisted hunting at Fongoli, but one that is not exclusive of those given previously, is that such hunting enables individuals who would be less likely to chase down larger vertebrate prey access to an energetically and nutritionally valuable food resource in a patchy savannah environment. Acquiring vertebrate prey via tool use at Fongoli supports the hypothesis that early hominins intensified their tool technology to overcome environmental pressures and that even the earliest hominins were probably sophisticated enough to fashion tools for hunting [12,14,20]. The behaviour of these chimpanzees demonstrates that hunting is less adult male-biased among our closest living relatives than previously believed [1,2] when tools are used, and emphasizes the need to take into account the range of behavioural variation within a species, specifically when findings are applied to attempts to understand evolutionary adaptations [43]. If tool use enabled early hominins to reduce the need for physical characteristics (i.e. greater size, strength) to achieve hunting efficiency, such sexual dimorphism ultimately becomes less important regarding prey acquisition. *Galago* hunting at Fongoli represents a high-energy, low-risk resource ([44], but see [40]) that members of various age–sex classes can take advantage of, similar to what is observed among tool-equipped human hunters [44]. Additionally, typical adult male chimpanzee arboreal hunting behaviour was unlikely to be characteristic of early bipedal hominins given the latter's anatomical differences with living apes [17]. With such anatomical changes in our lineage, tool use probably became increasingly important to hunting behaviour.

**Figure 5.**
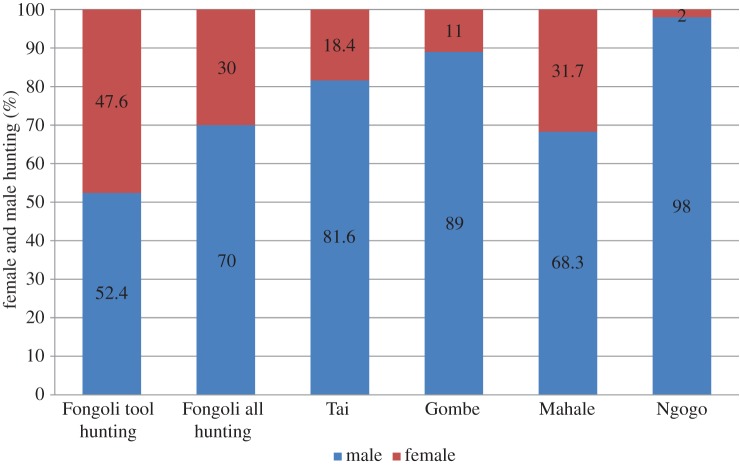
Hunting success among males and females at different study sites and at Fongoli [42,45–48]. Data are not controlled for differential observation of males and females among the different study sites.

Finally, we note that at least at one other site, Mahale, females also account for almost one-third of prey obtained [45], but such data receive less attention relative to male hunting behaviour (figure 5; [42,45–49]). Although two other cases of tool-assisted hunting have been reported at the Mahale site [46], given the rarity of this behaviour observed over the course of more than 50 years of study, these remain anecdotal and appear to be opportunistic rather than systematic, which may be best explained by relatively greater availability of vertebrate prey such as the red colobus monkey. These authors note, however, that such behaviour may be overlooked at Mahale and other sites given the lack of information on female chimpanzee behaviour compared to males [47]. We suggest that more detailed examination of female hunting behaviour can be informative in understanding the nature of sex differences in feeding and foraging, which can ultimately inform our understanding of extinct hominin behavioural ecology.

## Data accessibility

Data files containing raw data on Fongoli chimpanzee tool-assisted hunting behaviour analysed in this study can be found in the Dryad database (http://dx.doi.org/10.5061/dryad.5d1s0).
